# Upcycling Post-Consumer Plastic Waste into Electrospun Nanofibrous Separators for Sustainable Energy Storage

**DOI:** 10.3390/polym18182234

**Published:** 2026-09-13

**Authors:** Ayaulym Belgibayeva, Altynay Zhumabekova, Zhansaya Arkasheva, Nazym Makanova, Aitolkyn Uali, Aliya Mukanova, Zhumabay Bakenov, Sung-Soo Kim, Arailym Nurpeissova

**Affiliations:** 1 Institute of Batteries Limited Liability Partnership Kabanbay Batyr Ave. 53, Astana 010000, Kazakhstan; 2 Institute of New Materials and Energy Technologies Private Institution, Nazarbayev University, Kabanbay Batyr Ave. 53, Astana 010000, Kazakhstan; 3 Department of Chemistry, Lev Nikolayevich Gumilyov Eurasian National University, Kazhymukan Street 13, Astana 010000, Kazakhstan; 4 School of Engineering and Digital Sciences, Kabanbay Batyr Ave. 53, Astana 010000, Kazakhstan; 5 Graduate School of Energy Science and Technology, Chungnam National University, 99 Daehak-ro, Yuseong-gu, Daejeon 34134, Republic of Korea

**Keywords:** plastic waste upcycling, battery separators, electrospinning, lithium batteries, sustainable energy storage

## Abstract

The global economy is confronted by two parallel grand challenges: the unsustainable accumulation of plastic waste and the escalating demand for high-performance, sustainable energy storage technologies. This perspective operates at the nexus of these challenges and establishes a conceptual roadmap for upcycling post-consumer plastics into functional battery components. In particular, the potential of four widely available waste polymers, polyethylene terephthalate (PET), polyvinyl chloride (PVC), polystyrene (PS), and polymethyl methacrylate (PMMA), is examined for the fabrication of nanofibrous separator membranes through electrospinning. The distinct physicochemical characteristics of these polymers are analyzed in relation to key separator requirements, including porosity, electrolyte wettability, thermal stability, and ionic transport. Because studies employing waste-derived polymers remain limited, relevant research based on commercial polymers and alternative membrane fabrication approaches is also discussed to provide broader insight into structure-property-performance relationships. Environmental aspects related to recycling pathways, solvent systems, and life cycle considerations are also evaluated. Finally, major challenges such as feedstock variability, limited electrochemical validation, and scale-up limitations are identified, and future research directions are proposed. By integrating plastic waste upcycling with separator engineering, this perspective outlines a pathway toward circular and sustainable materials for next-generation energy storage systems.

## 1. Introduction

Driven by rapid global industrialization and expanding consumer markets, plastic production and consumption have increased dramatically over the past decades, resulting in the accumulation of large volumes of persistent waste in terrestrial and aquatic environments [1,2]. Current waste management strategies, including landfilling, incineration, and conventional recycling, remain insufficient to address this growing challenge [3,4]. As a result, plastic pollution has emerged as one of the most pressing environmental issues of the 21st century [5]. Global plastic production has exceeded 400 million metric tons annually, while only a small fraction, approximately 9%, is effectively recycled [1,6]. The remainder accumulates in landfills, leaks into ecosystems, or fragments into microplastics, leading to long-term ecological disruption and potential risks to human health [3,7]. Among the dominant plastic waste streams, polymers such as polyethylene terephthalate (PET), polystyrene (PS), polyvinyl chloride (PVC), and polymethyl methacrylate (PMMA) are particularly persistent due to their chemical stability and resistance to biodegradation. In addition, contamination with dyes, additives, and food residues often complicates conventional recycling processes [8,9]. These limitations highlight the urgent need for innovative recycling pathways capable of transforming waste plastics into value-added functional materials rather than merely recovering low-grade products.

In recent years, increasing attention has been directed toward the concept of upcycling, in which waste polymers are converted into advanced materials with enhanced functionality [10,11,12]. Various strategies have been explored to transform discarded plastics into porous carbons, nanofibrous membranes, catalysts, adsorbents, and polymer composites [13,14,15]. Such approaches aim not only to reduce environmental burdens but also to extend the lifecycle and utility of polymer resources. Among the available processing techniques, electrospinning has emerged as a particularly versatile method for converting polymer solutions into continuous nanofibrous structures with controllable morphology, high surface area, and interconnected porosity [16,17]. By applying a high electric field to a polymer solution or melt, ultrafine fibers with diameters ranging from tens of nanometers to several micrometers can be produced [18,19]. Importantly, electrospinning allows the incorporation of functional additives, surface modification, and structural engineering of membranes, enabling the fabrication of materials with tailored transport, mechanical, and surface properties [20,21,22,23,24,25]. Consequently, electrospun materials derived from waste plastics have been investigated for applications in filtration, adsorption, catalysis, sensing, and environmental remediation [26,27,28,29]. Despite these advances, the integration of waste-derived electrospun materials into energy storage technologies remains relatively limited [30,31,32,33,34].

Meanwhile, the global transition toward low-carbon energy systems has intensified the demand for efficient and sustainable energy storage technologies [35,36,37]. Rechargeable batteries, particularly lithium-ion batteries (LIBs), lithium-sulfur batteries (LSBs), and emerging sodium-ion systems, have become essential components of portable electronics, electric vehicles, and grid-scale storage [38,39,40]. While significant progress has been achieved through the development of advanced electrode materials and electrolytes, other cell components have received comparatively less attention. In particular, battery separators play a critical role in determining the safety, ionic transport properties, and long-term stability of electrochemical devices [41,42]. Separators serve as porous membranes that provide mechanical isolation between the cathode and anode, while simultaneously allowing for unhindered lithium-ion transport [43,44]. In conventional battery architectures, these separators are fabricated using polyolefin-based materials such as polyethylene (PE) and polypropylene (PP), which have been selected for their high chemical inertness and mechanical strength [45]. However, limitations including poor thermal dimensional stability, low affinity for polar electrolytes, and insufficient tolerance to elevated voltages and reactive species have been increasingly reported [46,47,48,49]. As battery systems evolve to meet the demands of high-power, high-temperature, and high-safety applications, it has become apparent that next-generation separators must be developed with enhanced multifunctionality and environmental compatibility [50,51,52].

**Figure 1:**
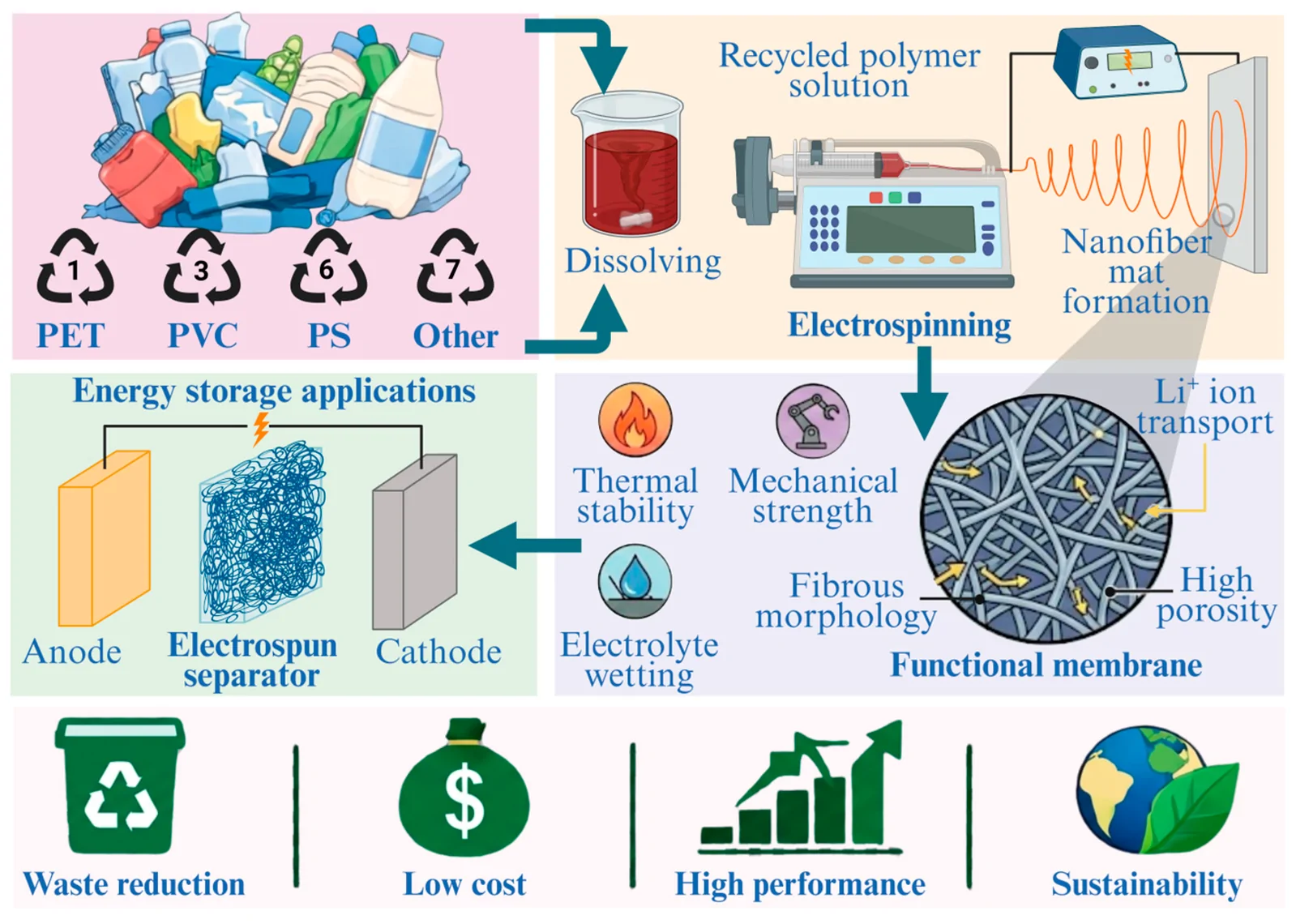
Schematic illustration of the circular upcycling of plastic waste into functional separators for advanced energy storage systems. Created in BioRender. Nurpeissova, A. (2026) https://BioRender.com/crw2jvm. Accessed on 4 March 2026.

In response, a variety of alternative polymers, including PET, PVC, PS, and PMMA, have been explored as components of advanced separator membranes or polymer electrolytes because of their favorable thermal stability, structural integrity, and tunable surface chemistry [53,54,55]. For example, PET-based membranes coated or blended with polymers like poly(vinylidene fluoride-co-hexafluoropropylene) (PVDF-HFP), or reinforced with ceramic fillers, have shown improved electrolyte wettability, thermal shrinkage suppression, and enhanced cycling performance [56,57,58,59], while PVC- and PMMA-containing membranes have demonstrated increased ionic conductivity and interfacial stability when plasticized, blended, or functionalized with inorganic additives [60,61,62,63,64]. Considering that many of these polymers are widely present in post-consumer plastic waste streams, their direct conversion into functional battery components represents an attractive opportunity to integrate polymer recycling with energy storage technologies. In this context, the upcycling of plastic waste into electrospun separator membranes represents a promising strategy that can simultaneously mitigate environmental pollution and enhance the sustainability of battery materials. Furthermore, electrospun nanofibrous separators possess several intrinsic advantages, including high porosity, interconnected pore networks, tunable surface chemistry, and improved electrolyte uptake [50,65]. These characteristics are beneficial for achieving efficient ion transport and stable electrochemical operation. Nevertheless, despite the growing interest in both polymer recycling and electrospun separators, only a limited number of studies have reported the direct fabrication of battery separators from post-consumer plastic waste. The conceptual framework for converting plastic waste into functional separator membranes for energy storage systems is illustrated in Figure 1.

In this perspective review, recent progress in upcycling plastic waste into electrospun separators for energy storage systems is critically examined, recognizing that research in this area remains limited. The fundamental design requirements for battery separators are first outlined, followed by a discussion of electrospinning principles, solvent selection, and process parameters tailored for the transformation of plastic waste into nanofibrous membranes. Structure–property–performance relationships that govern ionic conductivity, mechanical robustness, and interfacial stability are analyzed to understand how waste-derived materials can meet application demands. Given the limited number of studies, available electrochemical performance data from lithium-ion, lithium-sulfur batteries, and supercapacitors are reviewed to evaluate the practical advantages and challenges of waste-derived membranes. Future directions are proposed, focusing on process optimization, lifecycle assessment, standardization, and integration into emerging circular battery technologies. Ultimately, this manuscript differentiates itself from existing literature by addressing an emerging research direction at the absolute intersection of plastic waste upcycling and electrochemical energy storage. By evaluating the adaptability of post-consumer polymers (PET, PVC, PS, and PMMA) alongside green solvent chemistry and device-level performance, this work bridges traditionally independent domains into a cohesive roadmap for next-generation energy storage systems.

## 2. Separator Requirements and Electrospinning of Waste Plastics into Nanofibrous Membranes

Table 1 summarizes the key characteristics of an "ideal" separator in comparison to conventional PP separators (Celgard^®^ 2400, Asahi Kasei Group, Japan) with corresponding characterization techniques. Translating these requirements into practice depends on fiber-level control. In electrospinning, a high-voltage field draws a polymer solution into ultrafine fibers deposited as a nonwoven mat [66,67,68]; the resulting pore structure and fiber diameter can be tuned via solution viscosity, electric field strength, flow rate, and collector configuration [69]. This control is governed by solution and process parameters rather than by whether the polymer is virgin or reclaimed, which is what makes electrospinning relevant to plastic waste upcycling in the first place: PET, PVC, PS, and PMMA have each been electrospun into nanofibers for applications ranging from filtration to biomedical scaffolds [29,70,71,72] and more recently into battery-relevant membranes when derived from waste streams [30,31,32,33,34].

Whether that promise holds depends on polymer-specific electrospinnability and achievable membrane properties, which the following subsections examine in turn for each of the four polymers, assessed against Table 1 benchmarks; direct battery performance data, where they exist, are treated separately in Section 3 once membrane-level properties have been established.

### 2.1. Polyethylene Terephthalate (PET)

PET is the most extensively studied plastic waste source for electrospun membrane fabrication, owing to its favorable solubility, high intrinsic strength, and established solvent systems [73,74]. It is recovered primarily from beverage bottles, food containers, and textiles [75,76]; because these streams differ in intrinsic viscosity, prior thermal history, and additive content, they do not electrospin identically, a point developed further below.

The transition from bulk PET waste to functional nanofibers requires precise control over the process parameters. Table 2 outlines these optimized electrospinning parameters for PET and contrasts them with the requirements for other major plastic categories, providing a technical roadmap for the upcycling processes detailed throughout this section and the following subsections.

The synthesis of nanofibers via electrospinning begins with the preparation of a spinnable PET solution. Mechanical pretreatment steps, such as cutting the plastic waste into 5 × 5 mm^2^ pieces or smaller, are followed by dissolution in mixtures of strongly acidic or halogenated solvent systems under magnetic stirring at room temperature [70,77,78]. Once a homogeneous solution with adequate viscosity is achieved, electrospinning is carried out, resulting nonwoven mats with nanofiber diameters in the range of ~300 nm to 1 μm, forming porous membranes with interconnected morphology. Post-treatment involves drying at ambient conditions or under vacuum at 50 °C for several hours [78,79]. The membranes produced under these conditions consistently exhibit porosities above 80%, roughly double the 41% reported for Celgard^®^ 2400 (Table 1), specific surface areas between 10 and 25 m^2^ g^−1^, a fiber-morphology metric for which commercial separators do not typically report a directly comparable value. Fiber networks show thermal stability up to ~150 °C [58,80], directly meeting Table 1’s ideal-separator target (≥150 °C) and outperforming Celgard^®^ 2400, which shrinks at only 90 °C.

Mechanical performance is where waste-derived PET currently falls short of Celgard^®^ 2400 rather than exceeds it: reported tensile strengths range from 1 to 62.5 MPa, depending on fiber alignment, additive content, and drying conditions [81], below Celgard’s 70–140 MPa and, at the low end, below even the 15 MPa floor of Table 1’s ideal-separator target. Surface wettability follows a similar treatment-dependent pattern, though the available data are water contact angle (WCA) measurements rather than direct electrolyte wettability. Pristine PET shows WCA exceeding 120°, and mild surface oxidation, plasma treatment, or UV-ozone exposure introduce polar groups and lower the WCA to below 40° [82,83]. A low WCA indicates a more polar, higher-surface-energy fiber surface, the same underlying property Table 1’s “high surface energy/polarity” target refers to for electrolyte wettability, but WCA is a proxy measured against water, not the carbonate-based electrolytes used in real cells, so it should be read as suggestive of improved electrolyte affinity rather than as a direct measurement of it. Table 3 extends this comparison to membranes from PET and other waste streams reported in non-electrochemical studies, evaluated against the same Table 1 benchmarks.

### 2.2. Polyvinyl Chloride (PVC)

High production volume of PVC across construction, piping, flooring, and packaging makes it a major post-consumer waste stream [84], but it has long been regarded as difficult to electrospin due to its partial solubility, low thermal stability, and high additive content. Recent studies demonstrate that, with appropriate purification and solution preparation, waste-derived PVC can nonetheless serve as a viable precursor for functional nanofibrous membranes [85,86,87,88,89,90].

Although most early reports were based on commercial PVC powders, they provide important benchmarks for processing waste-derived PVC. Electrospinnable solutions typically contain 5–20 wt% PVC, depending on the desired fiber diameter and membrane morphology [91,92]. However, unlike commercial PVC powders, post-consumer PVC—particularly from piping or flooring—contains a substantial fraction of inorganic fillers (e.g., CaCO_3_), plasticizers, and stabilizers that can impair solubility and fiber formation. To address this, centrifugation-based purification has emerged as a practical and effective method.

**Table 3:** Summary of Electrospun Waste-Derived Membranes (Non-Electrochemical Studies). Data are included as indirect evidence of achievable membrane properties, not demonstrated separator performance, and are read against Table 1 benchmarks.

Source of Plastic	Membrane Characteristics	Original Study Context	Refs
PET bottle	Fiber diameter: 1 ± 0.19 μm; TS: 62.5 MPa; Young’s modulus: 1.39 GPa; toughness: 65.5 MJ m^−3^; shrinkage: 43% at 150 °C; Tg: 80.5 °C; Xc: 6.0%	Smoke filtration	[70]
PET bottle	Fiber diameter: 0.67 μm; thickness: 220.79 μm; porosity: 96.7 ± 0.05%; TS: 4.5 MPa; Maximum pore area: 27.28 µm^2^	Air filtration	[93]
No-dye PET fabric	Fiber diameter: 0.175 ± 0.07 μm; Young’s modulus: 194.5 ± 10.8 MPa; Tg: 72.7 ± 1.0 °C; Xc: 19.4 ± 0.2%		[79]
Red/blue-dye PET fabric	Fiber diameter: 0.240 ± 0.095 μm; Young’s modulus: 192.9 ± 7.5 MPa; Tg: 72.5 ± 0.8 °C; Xc: 19.8 ± 0.3%		[79]
PET bottle	Fiber diameter: 0.095 ± 0.037 μm; vapour permeability: 94%; breathability: 39.13 mm s^−1^; thickness: 25 μm; basis weight: 14 g m^−2^; TS: 2.7 ± 0.3 MPa; strain-at-break: 46 ± 9%	Filtration membrane (face mask alternative)	[28]
PET bottle	Fiber diameter: 0.1985 μm; wetting time: top surface—6.568 s, bottom surface—59.359 s; OMCC: water repellent; air permeability: 8.59 L min^−1^; maximum strength: 20 N; elongation value: 4.68 mm	Air filtration	[94]
PET bottle	Fiber diameter: 0.21 μm; TS: 42 MPa	Smoke filtration	[78]
PET bottle	Fiber diameter: 0.89 μm; Young’s modulus: 32.8 MPa; strain-at-break: 39.4%	Oil structuring	[77]
Recycled PET	Fiber diameter: 0.433 ± 0.11 μm;		[95]
PET bottle	Fiber diameter: 0.71 ± 0.249 μm; TS: 3.4 ± 0.1 MPa; strain-at-break: 273 ± 20%; roughness Ra: 3.4 μm; WCA: 137.6°	Oil-water separation	[96]
Recycled-PET	Fiber diameter: 0.15–0.43 μm; average pore area: 9.6 ± 1.1 × 104 nm^2^; average porosity: 19 ± 1%; WCA: 123.8 ± 6.8°; Xc: 46%; Tg: 92.5 ± 0.1 °C; TS: 1.8 ± 0.2 MPa; Young’s modulus: 36.3 ± 2.0 MPa; strain-at-break: 14 ± 2%		[97]
Waste PVC pipes	Fiber diameter: 0.443 ± 0.125 μm; thickness: 0.069 ± 0.002 nm; UTS: 3.36 MPa; WCA: over 100°; Young’s modulus: 197.46 MPa; porosity: over 65%	Air filtration	[90]
Waste PVC	Fiber diameter: 0.58 ± 0.42 μm	Water Treatment	[98]
Waste EPS	Fiber diameter: 1.33 μm; WCA: 123.3°; porosity: 79%	Oil–water separation	[71]
EPS food packaging waste	Fiber diameter: 1.008 ± 0.181 μm; WCA: ~137°; TS: ~ 18 MPa; UTS: ~11 MPa; Young’s modulus: ~700 MPa; porosity: ~18%	Air filtration	[99]
EPS craft waste	Fiber diameter: 1.183 ± 0.194 μm; WCA: ~136°; TS: ~14.8 MPa; UTS: ~10.5 MPa; Young’s modulus: ~500 MPa; porosity: ~36%	Air filtration	[99]
ESP instant noodle cup waste	Fiber diameter: 1.248 ± 0.223 μm; WCA: ~143°; TS: ~10.2 MPa; UTS: ~8 MPa; Young’s modulus: ~450 MPa; porosity: ~51%	Air filtration	[99]
ESP electronic packaging waste	Fiber diameter: 1.273 ± 0.222 μm; WCA: ~137°; TS: ~5.5 MPa; UTS: ~9 MPa; Young’s modulus: ~400 MPa; porosity: ~55%	Air filtration	[99]
Waste PS foam	Fiber diameter: 9.540 ± 0.387 µm; WCA: 119 ± 3°	Oil-water separation	[100]

Abbreviations: PET—polyethylene terephthalate; PVC—polyvinyl chloride; EPS—expanded polystyrene; TS—tensile strength; UTS—Ultimate tensile strength; WCA—water contact angle; Xc—crystalline content.

**Figure 2:**
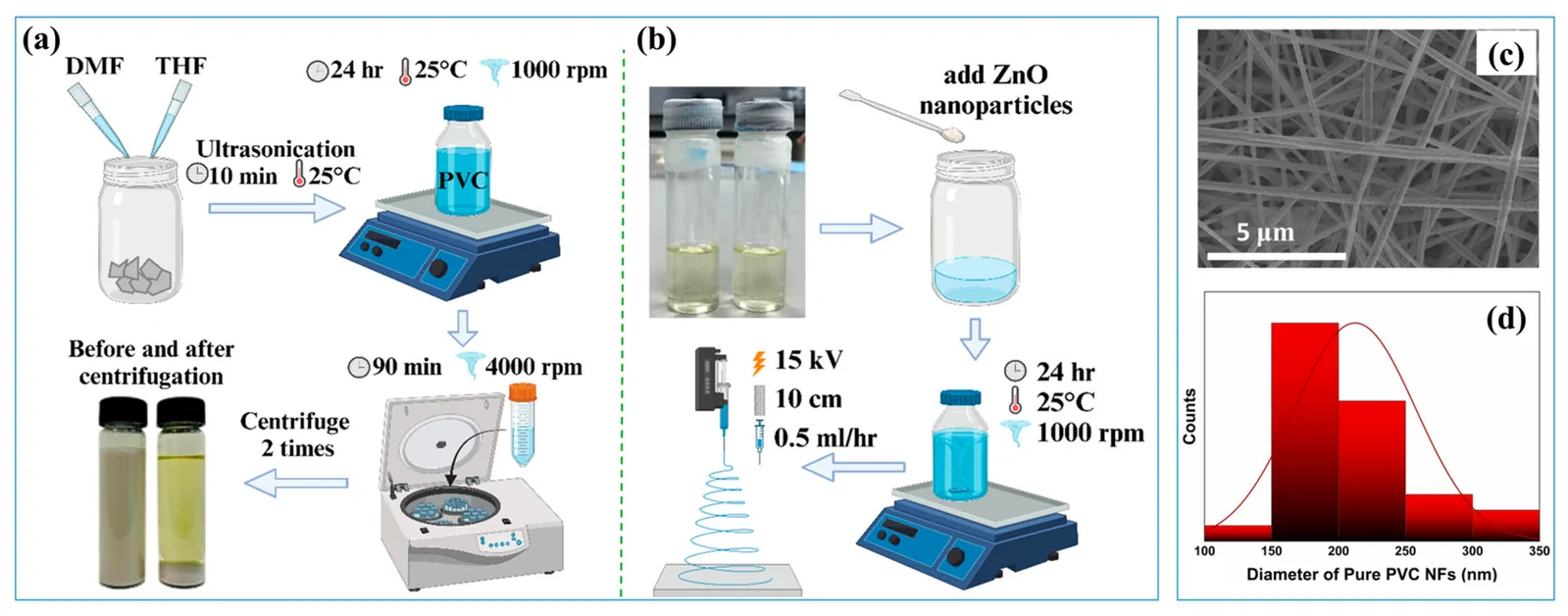
(**a**) Schematic design for recycling PVC polymer from waste PVC pipes. (**b**) The electrospinning process of PVC/ZnO NFs. (**c**) SEM images of pure PVC NFs and (**d**) Diameter distribution histograms. Reproduced from ref. [29] © 2025, Yavari et al.

For instance, Yavari et al. dissolved 2 g of PVC pipe waste (approximately 70 wt% polymer) in a THF:DMF mixture, followed by ultrasonication and 24 h stirring [29]. The solution was then centrifuged twice at 4000 rpm for 90 min, and the clear supernatant was used for electrospinning, as illustrated in Figure 2a. A similar approach was used by Amalia et al., who dissolved waste PVC in DMAc (10–30 wt%) and centrifuged the mixture at 6000 rpm for 60 min to remove insoluble impurities and additives [90]. These steps were crucial for reducing phase separation and nozzle clogging during electrospinning.

Following purification, PVC solutions can be electrospun under ambient conditions, as illustrated in Figure 2b. Resulting fiber diameters typically fall in the range of 300–800 nm, forming flexible, nonwoven mats, as shown in Figure 2c,d. Membranes derived from purified waste PVC show porosities of 60–75%, exceeding both Celgard^®^ 2400’s 41% and Table 1’s >40% ideal-separator threshold. Mechanical performance is more mixed: the Young’s modulus reported for waste PVC pipe-derived fibers, 197.46 MPa, sits close to Celgard’s 170 MPa and within Table 1’s 100–2000 MPa ideal range, but ultimate tensile strength for the same fibers, 3.36 MPa, falls far short of both Celgard’s 70–140 MPa and Table 1’s 15 MPa floor, a gap consistent with the wide tensile-strength variability already noted for PET and likely reflecting incomplete removal of plasticizers and fillers during purification.

Wettability of pristine PVC membranes tends to be low, with WCA often above 90° [101], a proxy measurement rather than direct electrolyte wettability, but one that places untreated PVC closer to the “poor” wettability of conventional PP/PE separators (Table 1) than to a high-surface-energy target. Surface modification or blending strategies are commonly employed to improve this parameter and to enhance interaction with polar environments. One of the unique advantages of PVC is its chemical modifiability via substitution of chlorine atoms on the polymer backbone: chemical modification, whether via chlorine substitution to graft polar groups [102], alkaline/acid activation to introduce hydroxyl or carboxyl groups, or incorporation of inorganic fillers (e.g., ZnO or TiO_2_) can enhance surface roughness and surface energy [24,25,89,91]. These modifications, though primarily demonstrated on commercial PVC powders, are expected to transfer to purified waste-derived material provided molecular weight and processing conditions are controlled.

Waste-PVC-based membrane development remains limited to a small number of reports, and further work is needed to optimize solvent systems, especially greener alternatives to DMF and DMAc, and to evaluate the scalability of purification methods such as centrifugation or filtration.

### 2.3. Polystyrene (PS)

Expanded polystyrene (EPS), widely used in packaging and insulation, represents one of the most voluminous and challenging forms of plastic waste due to its low density and high transportation cost [2]. Its chemical structure, based on aromatic styrene units, offers favorable solubility in a variety of organic solvents and high thermal stability, making it an attractive candidate for upcycling via electrospinning [103]. Although EPS has rarely been employed directly in electrochemical energy systems, it has been successfully processed into fibrous membranes using relatively mild and cost-effective methods.

Waste PS is typically washed, dried, and mechanically shredded before being dissolved in THF, DMF, DMAc, chloroform, acetone, their mixtures, or greener alternatives such as d-limonene [104,105]. The electrospinning parameters specified in Table 2 typically produce fibers with diameters ranging from 300 nm to several µm, as illustrated in.

As shown in, the WCA of EPS nanofibrous membranes increases from 106° to 153° as the fiber diameter decreases, indicating a transition from hydrophobic to superhydrophobic behavior. This trend is attributed to enhanced surface roughness and increased air trapping between individual fibers, which stabilizes water droplets and elevates the apparent contact angle; smaller fiber diameters and the presence of bead structures further intensify this effect. Because this superhydrophobicity is engineered for oil-water separation, where the same surfaces are typically oleophilic, its implication for wetting by carbonate-based battery electrolytes, which are structurally closer to oils than to water, is unclear from WCA alone and cannot be assumed to track the same direction. Specifically, EPS fibers with average diameters of 1889, 844, and 325 nm exhibited WCAs of 106°, 137°, and 153°, respectively, values that are higher than those reported for comparable electrospun systems such as PAN, PAN/FPU composites, and PCL fibers. On the other hand, the surface energy of PS is readily tunable via roughness and functionalization, as shown by waste-PS studies engineering it toward strong water repellency for fog-water harvesting [106] and oil-water separation [71]. Separator applications would need that same tunability redirected toward higher polarity instead; the MOF-incorporated adsorbent from waste PS foam reported by Thamer et al. [107] is the closest existing example, though it does not report a wettability value.

The porosity increases with increasing polymer concentration and fiber diameter, while the Young’s modulus strongly depends on fiber morphology, reaching 1564.3 MPa for beaded fibers, 989.3 MPa for wrinkled fibers, and 114.5 MPa for smooth fibers, values that bracket Celgard^®^ 2400’s reported modulus range and indicate that PS’s mechanical performance is not the limiting factor here.

### 2.4. Polymethyl Methacrylate (PMMA)

Post-consumer PMMA waste arises from signage, display sheets, and injection-molded parts [11,108]. In electrospinning research, virgin PMMA has been explored both as a membrane-forming polymer and, more recently, as a pore-forming agent in composite systems designed for thermal treatment [109,110]. Its favorable solubility in common organic solvents, clean decomposition behavior, and high glass transition temperature have positioned PMMA as a versatile component in electrospun membrane design [111,112]. On the other hand, no waste-derived PMMA has been electrospun and characterized for any separator-relevant property (porosity, wettability, mechanical strength) in the literature identified for this review. Electrospinning protocols from Table 2 have not yet been broadly tested on recycled PMMA at all. PMMA is therefore the least evidenced of the four polymers considered in this review, with no waste-derived electrospun membrane data of any kind, battery-relevant or otherwise, reported to date. Its established solubility, clean decomposition, and thermal stability suggest it is technically amenable to the same electrospinning and purification approaches demonstrated for PET, PVC, and PS, but this remains an extrapolation rather than a documented result. Advancing this line of work will require, at minimum, a first study electrospinning waste-derived PMMA and reporting the porosity, wettability, and mechanical properties needed to place it on the same evidentiary footing as the other three polymers in this review.

### 2.5. Other Plastics and Mixed Waste

Several additional post-consumer plastics, including acrylonitrile butadiene styrene (ABS), polylactic acid (PLA), and polyvinyl butyral (PVB), have also been electrospun from recycled feedstocks, though almost entirely for air or water filtration rather than battery applications. Waste ABS blended with PVP has been shown to reverse the native WCA of the polymer (132° to 53° for a 23:5 ABS:PVP blend) [113], a water-based proxy rather than a direct electrolyte-wettability measurement, as noted for the polymers above; recycled PLA has been carbonized into porous electroactive carbon nanofibers [114], and windshield-derived PVB has been electrospun into cohesive, mechanically robust mats [115]. None of these has been tested as a battery separator, and none is carried further in this review; they are noted here only to indicate that the waste-to-nanofiber approach developed for PET, PVC, PS, and PMMA is not unique to those four polymers.

### 2.6. Comparative Assessment of Waste-Derived Polymers for Separator Applications

A comparative examination of electrospun membranes derived from various plastic wastes reveals clear trends in material behavior, processing compatibility, and functional potential. Despite differences in origin and chemical structure, most waste polymers investigated—including PET, PVC, PS, PMMA—can be transformed into nanofibrous mats using broadly similar electrospinning conditions, although solvent selection must be tuned to balance polymer solubility with environmental and safety considerations.

As for the degree of required pre-processing, it varies widely. PET and PMMA waste typically dissolve cleanly with minimal purification, whereas PVC often demands centrifugation or sedimentation steps to remove inorganic fillers. PS, though chemically inert, is highly soluble and straightforward to spin.

Table 4 summarizes this comparison directly against the criteria most relevant to separator selection, distinguishing established evidence from extrapolation rather than assigning a single composite score. PET is currently the most complete case, strong on electrospinnability and thermal stability, but weak and inconsistent on mechanical strength. PVC and PS each show one clear structural advantage (porosity and modulus, and mechanical performance, respectively) alongside one clear open problem (tensile strength, and wettability direction, respectively). PMMA remains largely unevidenced as a waste-derived separator candidate and is better understood, at present, as a subject for future first studies than as a developed option alongside the other three. A parallel comparison of demonstrated battery performance follows once that evidence is presented in Section 3.

Table 4, however, presents each cell as though it were a stable property of the polymer, which the underlying data do not fully support. A recurring constraint across all four polymers, and one easy to understate when reporting a single representative value, is that post-consumer feedstock is not a uniform input in the way virgin polymer pellets are; source stream, prior processing history, and residual additive content all vary and, in the studies reviewed above, are not always reported in enough detail to compare across papers directly. For PET, this has been directly demonstrated: electrospun nanofibers from postconsumer polyester textiles differ structurally from those derived from PET bottles [79], reflecting differences in intrinsic viscosity, molecular weight distribution, and prior thermal history between the two source streams. For PS, the same effect is visible within a single dataset in Table 3: four EPS feedstocks reported by Lima et al. [99], sourced from food packaging, craft waste, instant-noodle-cup waste, and electronic packaging, span porosity from 18% to 55% and tensile strength from 5.5 to 18 MPa under nominally the same processing route, variation attributable to feedstock source rather than method, and wider than Table 4 entry for PS alone can convey. For PVC, the dominant variability is compositional rather than structural: post-consumer material from piping or flooring carries a substantial and source-dependent fraction of inorganic fillers, plasticizers, and stabilizers (Section 2.2), which is why purification protocols, rather than solvent chemistry, are the main processing lever reported in that literature. For PMMA, feedstock variability cannot yet be assessed at all, since no waste-derived electrospinning study exists to characterize it, consistent with the “No data” entries already shown for it in Table 4.

Two practical consequences follow. A reported property value for a given polymer, tensile strength, porosity, or wettability, should be read as a value for that specific feedstock rather than a fixed property of the polymer, unless the source stream is specified; and this is a gap in reporting practice as much as a materials limitation, since none of the studies discussed above characterized intrinsic viscosity, residual additive content, or thermal history systematically alongside membrane performance, which limits how far results from one feedstock can be generalized to another.

## 3. Electrochemical and Chemical Compatibility of PET, PVC, PS, and PMMA for Battery Separator Applications

Section 2 addressed the physical membrane properties of waste-derived electrospun materials, porosity, wettability, mechanical strength, and thermal stability, benchmarked against Table 1. Chemical stability and compatibility with specific battery chemistries are separate and equally necessary questions, and ones this section addresses directly. Studies combining both waste-derived sourcing and electrospinning, the specific focus of this review, turn out to be limited even for PET, the most extensively evidenced polymer here, and sparse or entirely absent for PVC, PS, and PMMA. As a perspective piece, the aim of this review is to assess the feasibility of that combination rather than report only where it has already been achieved, so this section reviews all available sources bearing on electrochemical and chemical compatibility, even where a given study did not use electrospinning or waste-derived material: each still demonstrates what the material itself is electrochemically capable of and supports extrapolation toward the combined waste-derived, electrospun case even where that combination has not itself been tested. Table 5 summarizes the preparation methods, cell configuration, and electrochemical performance of the PET-, PVC-, PS-, and PMMA-based separator and electrolyte studies identified for this review; several are gel electrolytes, phase-separated films, or particle-coated composites rather than electrospun fibers, and several use commercial rather than waste-derived material. The following subsections carry these distinctions through explicitly, benchmarked throughout against the commercial separator values established in Table 1.

### 3.1. PET-Based Membranes

Of the four polymers considered in this review, PET has the most extensive electrochemical evidence. Al_2_O_3_/PVDF-HFP-coated commercial PET reaches a 5.5 V electrochemical window against 4.6 V for PP [116], SiO_2_-functionalized PVDF-HFP/PET/PVDF-HFP achieves ~20% higher capacity retention than PE after 200 cycles at 55 °C [117], and PVDF-reinforced PET nonwoven sandwich separators show lower interfacial resistance than Celgard^®^ 2400 [81]. Two further commercial-PET studies (Table 4) report favorable membrane properties: a blade-coated PET@PVA@SiO_2_ separator with 306.6% electrolyte uptake [118], and a solution-cast PET gel-polymer electrolyte with 4.08 × 10^−4^ S cm^−1^ ionic conductivity [119]. Several of these also report ionic conductivity at or above the >10^−3^ S cm^−1^ ideal-separator target in Table 1 and the 6.5 × 10^−4^–1.15 × 10^−3^ S cm^−1^ range reported for Celgard^®^ 2400, for example, 8.36 mS cm^−1^ for the PET/PVDF hot-pressed membrane [120], establishing that PET as a material is electrochemically compatible with standard LIB electrolytes and cathode chemistries at a voltage range, ionic conductivity, and cycling depth relevant to practical cells.

Only one study in this broader body of work is both electrospun and uses commercial PET directly as the spun fiber: Hao et al. produced electrospun commercial-PET membranes with high porosity (89%) and an ionic conductivity of 2.27 × 10^−3^ S cm^−1^ [30] (Table 4), above both the Celgard^®^ 2400 and Table 1 ideal-separator thresholds, establishing that PET fiber mats formed by electrospinning specifically, not just PET fabric generally, are electrochemically viable.

Two studies extend this to genuinely waste-derived, electrospun PET with electrochemical-cell testing, and both speak directly to the wettability and mechanical limitations flagged for waste-derived PET generally. Tsang et al. electrospun PET/AlN composite separators from recycled water bottles, achieving high porosity (~69%) and exceptional electrolyte uptake (~522%), resulting in a reversible capacity of 238.2 mAh g^−1^ after 100 cycles at 0.5C in NCM 523||Li system [33]. The membrane with 5 wt% AlN demonstrated strong rate capability, retaining ~208.2 mAh g^−1^ at 1C and ~179.2 mAh g^−1^ at 2C. The improved performance was attributed to the synergetic effect of AlN’s thermal conductivity and dendrite suppression properties, along with the high surface area of the electrospun architecture. The WCA they reported, 135°, poor by any wettability standard, sits alongside an electrolyte contact angle of ~0°, near-perfect electrolyte wetting on the same fiber. This is a direct experimental confirmation of the earlier caution that WCA does not reliably predict electrolyte wettability.

Ramakrishnan et al. went further, electrospinning neat waste PET bottles with no functional filler at all, then testing the resulting membrane as a combined separator-electrolyte in both LiMn_2_O_4_||Li and Na_2/3_Ni_1/3_Mn_2/3_O_2_||Na cells [34]. The ionic conductivity, ~10^−3^ S cm^−1^, sits at the ideal-separator threshold in Table 1, the electrolyte contact angle of 0° directly outperformed the 55–56° reported for commercial PP within the same study, and mechanical performance was reported as comparable to Celgard^®^ 2400, though as a breaking load and strain percentage rather than the MPa tensile strength used elsewhere in this review, so it cannot be placed precisely within the wide 1–62.5 MPa range noted for waste-derived PET generally. Both studies matched Whatman glass-fiber separator performance in their respective cells.

Taken together, these results establish that chemical and electrochemical compatibility of PET with standard LIB, and, per Ramakrishnan et al., Na-ion metal-battery, chemistries are well established at the material level, and the two waste-derived, electrospun studies confirm this compatibility survives the transition to post-consumer feedstock and the target fabrication method.

### 3.2. PVC-Based Membranes

No study identified for this review reports a waste-derived, electrospun PVC membrane tested as a battery separator. What exists instead is a body of commercial-PVC electrolyte and membrane research establishing PVC as an electrochemically viable material, summarized below.

While most studies to date have focused on cast films, composite systems incorporating commercial PVC—such as PVC–PEO, PVC–PAN, and PVC–PMMA—have demonstrated room-temperature ionic conductivities in the range of 10^−4^–10^−3^ S cm^−1^ when doped with lithium salts and plasticizers [122,123,124], at the lower end of or below the >10^−3^ S cm^−1^ ideal-separator target in Table 1 and the 6.5 × 10^−4^–1.15 × 10^−3^ S cm^−1^ range reported for Celgard^®^ 2400. These systems also exhibit tunable mechanical properties through fillers like SiO_2_ or TiO_2_, and PVC–PMMA blends demonstrate that plasticizer-rich and PVC-rich domains can be engineered to separate ion-transport and mechanical roles within one membrane [124], a design principle not yet tested in electrospun, waste-derived form.

The closest analogue to what a waste-derived, electrospun PVC separator would need to demonstrate comes from a commercial PVDF-PVC electrospun composite, which reaches ionic conductivities of up to 2.25 × 10^−3^ S cm^−1^ at 25 °C, above Table 1 and Celgard^®^ 2400 thresholds, high porosity (~87%), excellent electrolyte uptake (~291 wt%), and good cycling stability over 50 cycles (90.1% retention) in LiFePO_4_-based cells confirmed the potential of such membranes for LIB applications [60]. The reduction in crystallinity due to amorphous PVC incorporation promoted lithium-ion mobility, enhancing both ionic conductivity and interfacial stability. This remains a single study, and has not been repeated on waste feedstock.

Further afield, commercial-PVC-based proton exchange membranes (PEMs) for high-temperature fuel cells and vanadium redox flow batteries (VRFBs) demonstrate the same chlorine-substitution chemistry flagged in Section 2.2 as a wettability fix, applied instead to ion-selective functionalization [21,102,125,126,127]. Imidazolium-grafted PVC membranes achieve higher ion selectivity than Nafion 115 in VRFB testing [125], and PVC functionalized with 1-(3-aminopropyl)imidazole reaches proton conductivity exceeding 0.26 S cm^−1^ at 180 °C under anhydrous conditions, with good mechanical integrity and wettability [21]. These are a different battery chemistry and a different ion than the LIB/LSB focus of this review, but they demonstrate the same modification route at a more advanced stage than anything reported for PVC in a LIB context.

### 3.3. PS-Based Membranes

No study identified for this review reports a waste-derived, electrospun PS membrane tested as a battery separator. Two commercial-PS studies, one supercapacitor and one battery, establish the electrochemical potential of the material.

**Figure 3:**
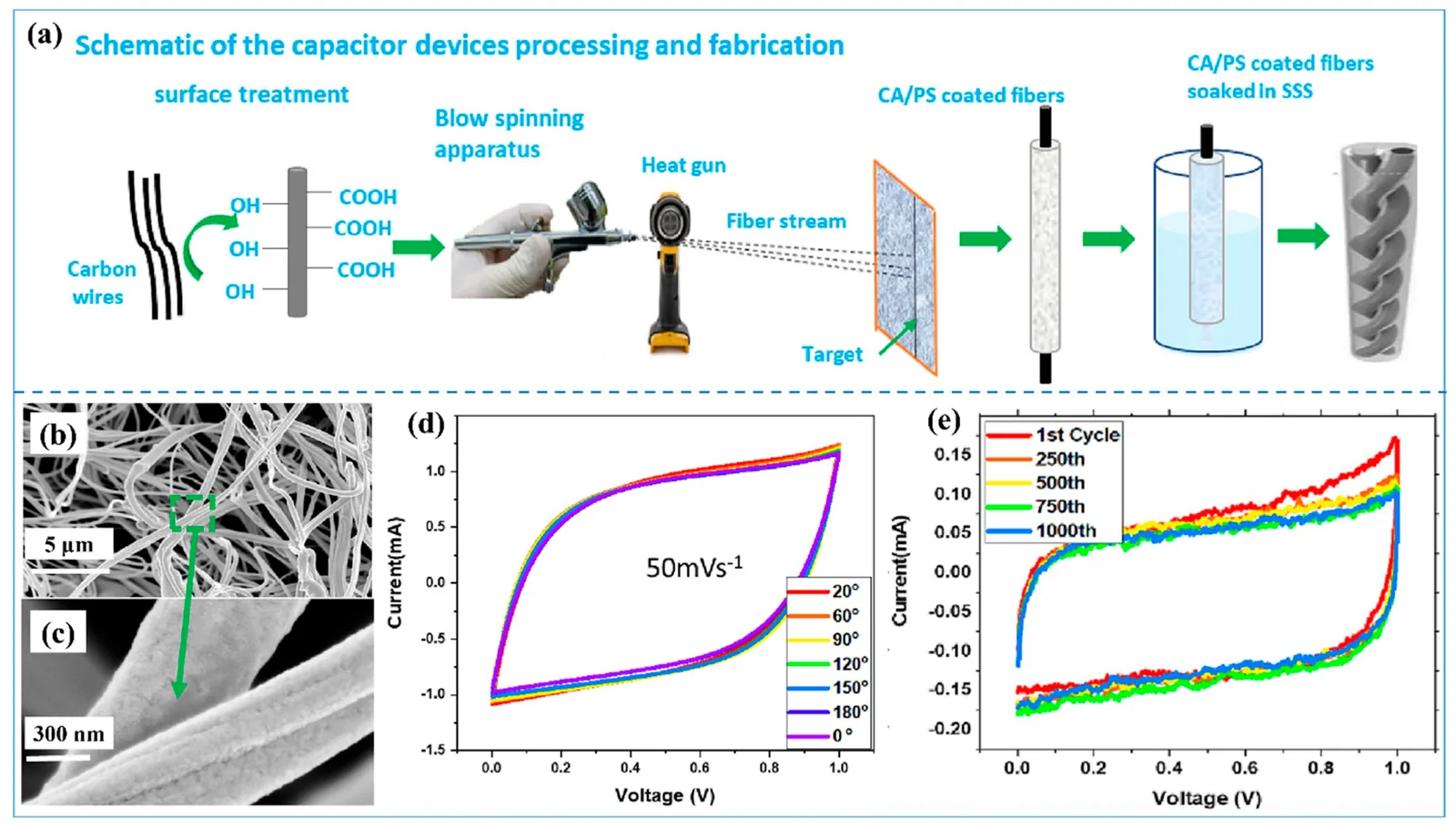
(**a**) Schematic illustration of cellulose acetate-based composite separator synthesis, (**b**,**c**) high-magnification SEM image of the composite separator, (**d**) comparison of CVs recorded at different angles, (**e**) cyclic stability of the device. Reproduced with permission from ref. [54], © 2023 Rafique et al. Published by Elsevier B.V.

In one of the few electrochemical studies, Rafique et al. fabricated a fibrous CA–PS composite separator using blow spinning and demonstrated its utility in fiber-shaped symmetric supercapacitors (Figure 3a–c) [54]. While CA contributed to electrolyte uptake, PS enhanced structural robustness and stability under mechanical stress (Figure 3d). The device maintained 95% capacitance retention after 1000 cycles (Figure 3e), indicating that PS can function as a reinforcing framework in composite membranes, though its direct role in ionic transport was minimal.

A second study demonstrates PS directly in a LIB separator role. Liu et al. electrospun PVDF/PS hybrid fibers, with ionic conductivity increasing alongside PS content, up to 1.58 mS cm^−1^ at 20 wt% PS, attributed to PS increasing the amorphous region of the PVDF matrix, above Table 1 ideal-separator threshold and the Celgard^®^ 2400 range [128]. Tensile strength rose to 15.9 MPa with increasing PS content, and non-flammability was contributed by the PVDF component, but porosity, electrolyte uptake, and average pore size all decreased over the same range, a direct trade-off between contribution of PS to conductivity and strength on one hand and porosity and uptake on the other. This is the strongest single electrochemical data point for PS in this review, though it uses commercial PS, PVDF is the majority phase governing ion transport, and it has not been repeated on waste-derived material.

Taken together, these two studies show PS is electrochemically viable both as a reinforcing composite component and, blended with PVDF, as a primary separator material with ionic conductivity exceeding Table 1 target. Neither uses waste-derived PS; extending either result to waste-sourced material is what would connect the evidence of this section to membrane-property findings of Section 2.3.

### 3.4. PMMA-Based Membranes

No study identified for this review reports a waste-derived, electrospun PMMA membrane tested as a battery separator, consistent with finding of Section 2. that no waste-derived PMMA has been electrospun and characterized at all. What exists is commercial-PMMA evidence, and none of it is fibrous: a particle-coated film, a crosslinked gel, and a phase-separated film, summarized below.

In Li–S systems, PMMA has been successfully applied as a polysulfide-suppressing barrier. Deng et al. reported a double-layered separator composed of a PP matrix and an arrayed PMMA microsphere layer [61]. This configuration demonstrated an initial discharge capacity of 1100 mAh g^−1^ at 0.1 mA cm^−2^, surpassing the performance of a conventional PP separator (948 mAh g^−1^). The PMMA microspheres physically constrained the diffusion of polysulfides while also chemically interacting with them via ester group adsorption, confirmed by FTIR analysis. The separator also exhibited improved electrolyte affinity and promoted Li^+^ ion diffusion due to the swelling behavior of PMMA in ether-based solvents.

In LIBs, crosslinked PMMA-based gel polymer electrolytes reach high ionic conductivities. Hosseinioun and Paillard developed an in situ crosslinked PMMA gel incorporating LiTFSI and LiBOB salts [64]. The resulting self-standing gel delivered an ionic conductivity of 3.9 mS cm^−1^ at 20 °C, well above Table 1 ideal-separator threshold and the Celgard^®^ 2400 range, and enabled stable cycling of NMC111||graphite cells over several hundred cycles. The low glass transition temperature and high liquid uptake of PMMA contributed to high lithium-ion mobility without compromising mechanical cohesion.

Similarly, Liu et al. prepared PVDF/PMMA blend membranes using a non-solvent-induced phase separation method and tested them in LIBs [62]. The optimal blend (PVDF:PMMA = 6:4) showed lowered electrolyte contact angle, an ionic conductivity of 2.18 mS cm^−1^, again above Table 1 threshold, and delivered a reversible capacity of 130.7 mAh g^−1^ after 200 cycles at 1C with a LiFePO_4_ cathode. Enhanced rate capability (133.3 mAh g^−1^ at 4C) and compatibility with lithium metal were also observed, indicating that PMMA can mitigate interfacial resistance and maintain structural integrity under high-rate cycling.

Ceramic fillers further raise conductivity in PMMA electrolytes: SiO_2_ nanoparticles increased PMMA–LiCF_3_SO_3_ conductivity by over two orders of magnitude (from 1.16 × 10^−6^ to 7.3 × 10^−5^ S cm^−1^), while improving thermal stability and interfacial contact [129]. In the same way, Romero et al. later showed that LLTO–PMMA nanocomposite electrolytes exhibited room-temperature conductivities above 1 × 10^−4^ S cm^−1^, confirming the role of ceramic additives in promoting ion dissociation and polymer chain mobility [130], though both remain below Table 1 threshold and neither is a fibrous membrane.

Taken together, these results establish PMMA as electrochemically compatible with LIB and LSB chemistries at the material level. None of this evidence is fibrous or waste-derived, and combined with Section 2.4’s finding that no waste-derived PMMA has been electrospun at all, PMMA remains the polymer furthest from the specific case this review evaluates: an electrospun, waste-derived membrane has yet to be attempted, let alone tested electrochemically.

## 4. Sustainability Considerations, Life-Cycle Perspectives, and Technological Challenges

Electrospun membranes produced from recycled plastics have found broad utility in applications such as filtration, packaging, and biomedical scaffolds, where their high surface area and tunable microstructure offer clear advantages. Section 2 and Section 3 of this review examined how much of that utility transfers to battery separators specifically. Some properties transfer directly: porosity and thermal stability advantages of PET over Celgard^®^ 2400 (Section 2.1) held up in the two waste-derived electrochemical studies identified (Section 3.1). Others do not transfer as cleanly as assumed: WCA, the standard metric in filtration and adsorption studies, did not predict electrolyte wettability for waste-derived PET (Section 3.1). No waste-derived electrochemical evidence exists for PVC or PS despite each having established filtration- or adsorption-relevant membrane properties (Section 2), and PMMA lacks even that: no waste-derived PMMA membrane of any kind, electrochemical or otherwise, has been reported (Section 2.4). The sustainability case for upcycled separators therefore currently rests almost entirely on PET, with the other three polymers still awaiting the specific waste-derived, electrospun, battery-tested combination this review has evaluated throughout.

Beyond immediate technical performance, electrospun membranes from post-consumer plastics offer a compelling pathway to integrate circular economy principles into energy storage technologies by transforming waste into high-value components. However, evaluating the true sustainability of this approach requires a critical shift from preliminary considerations to a rigorous, systems-level Life Cycle Assessment (LCA) and Techno-Economic Analysis (TEA). A central question in this domain—frequently raised in lifecycle validation—is whether the cumulative environmental and economic burdens of electrospinning waste plastics surpass those of manufacturing virgin polymer membranes or utilizing traditional mechanical recycling methods. To evaluate this trade-off quantitatively, the net environmental impact (ΔE_net_) of an upcycled plastic separator must be modeled using a systematic boundary equation that accounts for mass and energy balances across the entire processing lifecycle (Equation (1)):

ΔE_net_ = E_recovery_ + E_purification_ + E_solvent_ + E_spinning_ + E_modification_ − E_credit_ (1)

where

- E_recovery_ is the energy and environmental burden of collecting and sorting the post-consumer plastic waste.
- E_purification_ represents the chemical and thermal energy required to remove additives, plasticizers, and contaminants from the waste stream.
- E_solvent_ is the upstream environmental footprint of solvent synthesis combined with the energy required for post-process solvent recovery or emissions abatement.
- E_spinning_ represents the electrical energy consumption of the high-voltage, high-pressure electrospinning apparatus per kilogram of functional membrane.
- E_modification_ represents the additional energy and chemical burden of post-spinning functionalization steps, such as chlorine-substitution grafting, sulfonation, plasma treatment, or filler incorporation, required to bring a waste-derived membrane to separator-relevant performance targets.
- E_credit_ is the environmental displacement credit gained by diverting plastic waste from landfills and avoiding the synthesis of equivalent virgin petroleum-based resins.

For the upcycling pathway to be environmentally justified, the net footprint must be lower than that of the incumbent technologies (ΔE_net_ < ΔE_virgin_). Among the various plastics studied, PET is the only polymer in this review with demonstrated separator potential to weigh against its life-cycle profile. LCAs indicate that PET derived from consumer waste retains up to 70% of the mechanical performance of newly synthesized resin while reducing energy consumption by up to 84% during initial material recovery (E_recovery_) [131]. PVC, although more challenging due to its chlorine content and thermal sensitivity, offers inherent flame-retardant properties beneficial for battery safety [84], a property that would need to be realized in an actual waste-derived PVC separator, which Section 3.2 found has not yet been demonstrated, for this life-cycle advantage to be realized in practice.

However, the closed-loop environmental benefits of both polymers are frequently compromised during the fabrication phase (E_solvent_ + E_spinning_). Lab-scale electrospinning of waste PET and PVC heavily relies on hazardous, halogenated solvents (e.g., TFA, HFIP, or THF) and high-voltage processing. When accounting for the upstream environmental burdens of toxic solvent synthesis and the immense energy required for absolute solvent recovery, the net carbon footprint and toxicity indices of these waste-derived membranes can paradoxically exceed those of virgin, petroleum-based polymer membranes manufactured via conventional phase inversion. Green-solvent alternatives exist, but unevenly across the four polymers: d-limonene has already replaced hazardous solvents for waste EPS electrospinning specifically [104,105], and γ-valerolactone (GVL) and Cyrene are validated PVC dissolution solvents in recycling contexts [85], though neither has yet been demonstrated in electrospinning waste PVC rather than dissolution/precipitation recycling. No green-solvent system has been demonstrated for waste PET or PMMA, meaning the solvent-burden problem described above remains unresolved for the polymer with the strongest separator evidence in this review.

Furthermore, when contrasted with traditional mechanical recycling—which downcycles plastics into low-value structural materials via energy-efficient melting and extrusion—the electrospinning pathway demands significantly higher energy inputs (E_spinning_) per kilogram of functional material. To justify this increased thermodynamic and chemical expenditure, the upcycled plastic must deliver a demonstrable performance premium (e.g., enhanced capacity retention, dendrite mitigation, or safety profiles in LIB, LSB, or Na-ion configurations) that extends the operational lifespan of the battery system, thereby amortizing its processing footprint over the device’s total life cycle.

To mitigate these trade-offs and ensure that ΔE_net_ remains net-negative, current research is pivoting toward scalable, energy-efficient spinning techniques like needleless or centrifugal electrospinning. These methodologies reduce the high-voltage and high-pressure requirements typical of conventional needle setups while maximizing throughput, effectively suppressing both E_solvent_ + E_spinning_.

No study identified for this review tests cycling beyond 300 cycles, or reports whether the feedstock variability documented in Section 2.6 propagates into cell-level reproducibility. Existing work remains concentrated on membrane fabrication and structural characterization rather than long-term electrochemical stability or degradation under real-world cycling conditions [68].

Ultimately, to transition upcycled separators from laboratory concepts to industrially viable components, prospective LCA and TEA must be integrated directly into the material design phase rather than treated as retrospective checkpoints. This requires strict, quantitative accounting of material and energy inputs, solvent impacts, emissions, and end-of-life scenarios [132,133,134]. A systems-level approach that combines scalable fabrication, green chemistry, and life-cycle metrics will be essential to ensure that upcycled separators are not only technically viable but also environmentally responsible in practice [135].

## 5. Outlook: Toward Circular and High-Performance Separator Materials

The transformation of plastic waste into electrospun separator membranes has been recognized as a promising approach to simultaneously address the challenges of polymer pollution and sustainable energy storage. While encouraging progress has been achieved—most notably with PET—many waste-derived polymers remain underutilized in electrochemical systems. Although functional membranes based on PVC, PS, and PMMA have been explored in adjacent fields, their electrochemical performance in energy storage devices has yet to be comprehensively demonstrated.

Through electrospinning, precise control over fiber morphology, porosity, and membrane architecture can be achieved. When applied to recycled polymers, this method has enabled the fabrication of membranes with enhanced electrolyte uptake, improved thermal stability, and tunable mechanical performance. However, to fully realize the potential of these materials, challenges related to feedstock heterogeneity, electrolyte compatibility, thermal shrinkage, and scalability must be systematically addressed. Waste-derived PET membranes have been shown, in the two studies identified in Section 3.1, to match commercial glass-fiber separator performance in electrochemical cell testing and to exceed commercial PP on specific measured metrics, including electrolyte contact angle and ionic conductivity. PVC, PS, and PMMA have each demonstrated promising electrochemical behavior when appropriately functionalized or blended, but exclusively on commercial rather than waste-derived material (Section 3.2, Section 3.3 and Section 3.4); no waste-derived electrochemical evidence exists yet for any of the three.

A future vision in which separators are derived from waste, processed through environmentally responsible methods, and integrated into high-performance battery cells has been increasingly supported by early findings. To move toward this goal, several actions must be prioritized. Electrochemical testing protocols for waste-derived membranes should be standardized, with separators systematically benchmarked against commercial analogues. Scalable and less environmentally intensive electrospinning methods should be developed using recoverable or bio-based solvent systems tailored to post-consumer polymers. Furthermore, a comprehensive life-cycle and TEAs should be conducted to evaluate net environmental and economic benefits across the separator value chain.

Broader adoption of these materials will depend not only on technical feasibility but also on cross-sector collaboration. Engagement from materials scientists, battery engineers, recycling industry stakeholders, and regulatory bodies will be essential. Only through such interdisciplinary efforts can the deployment of sustainable separator technologies be accelerated.

To support this transition, several research directions should be actively pursued. Direct electrochemical validation of waste-derived membranes in full-cell LIB, LSB, and Na-ion configurations must be undertaken, as long-term performance data remain limited. Systematic comparisons between pristine and recycled polymers should be conducted to assess the effects of degradation, additives, and contaminants on separator behavior and reliability.

Functionalization techniques that enable polarity tuning, dendrite mitigation, or polysulfide blocking should be adapted for recycled substrates. Post-spinning strategies—such as plasma activation, surface grafting, or incorporation of catalytic coatings—may offer viable pathways to enhance electrochemical performance. Hybrid membrane architectures, where recycled fibers serve as structural frameworks combined with functional layers, should also be explored to decouple mechanical and transport roles.

In parallel, green solvent systems, solvent recovery strategies, and closed-loop fabrication processes must be developed to reduce the environmental burden of electrospinning. The end-of-life behavior of waste-derived separators should be evaluated within standard battery recycling processes to ensure compatibility with current recovery protocols.

Finally, the establishment of standardization frameworks for characterization, quality control, and safety certification will be critical. Without clear performance benchmarks and industrial acceptance criteria, the integration of recycled separator materials into mainstream battery production will remain limited.

In summary, waste-derived electrospun membranes have been positioned as a next-generation solution for eco-conscious battery manufacturing. If functional requirements can be met through structural design and process innovation, these separators may play a defining role in advancing both energy storage performance and environmental responsibility.

## Figures and Tables

**Table 1:** Key Characteristics of Conventional Separator and an Ideal Battery Separator with Corresponding Characterization Techniques.

Parameter	Conventional Separator Celgard^®^ 2400 Monolayer PP Membrane	Ideal Characteristic	Characterization Technique
Porosity	41%, uniform	High (>40%), uniform	Mercury porosimetry, N_2_ adsorption–desorption analysis
Pore Size	0.03–0.5 μm	Narrow (<1 μm), well-controlled	Scanning electron microscopy, capillary flow porometry
Electrolyte Wettability	Poor	High surface energy/polarity	Contact angle measurement
Ionic Conductivity	From 6.5 × 10^−4^ to 1.15 × 10^−3^ S cm^−1^	>10^−3^ S cm^−1^ with liquid electrolyte	Electrochemical impedance spectroscopy
Mechanical Strength	Tensile: 70–140 MPa Puncture: 340–450 g	Tensile: 15–150 MPa Puncture: >300 g	Tensile and puncture testing
Young’s Modulus	0.17 GPa	0.1–2 GPa (application-dependent)	Tensile testing (stress–strain curve)
Thermal Stability	2.3–5.0% shrinkage at 90 °C	Stable ≥150 °C, no shrinkage	Thermogravimetric analysis, differential scanning calorimetry
Chemical Stability	Inert to electrodes and electrolytes	Inert to electrodes and electrolytes	X-ray photoelectron spectroscopy, Fourier Transform Infrared spectroscopy, chemical soaking
Thickness	25 μm	Thin (10–30 μm)	Micrometer, cross-sectional scanning electron microscopy
Dimensional Uniformity	Consistent	Consistent across large area	Optical microscopy, scanning electron microscopy
Electrochemical Stability Window	Up to 4.5 V vs. Li/Li^+^ (practical)	Up to 4.5–5.3 V vs. Li/Li^+^	Linear sweep voltammetry, cyclic voltammetry

**Table 2:** Solution preparation and electrospinning parameters for different plastic types.

Plastic Type	Solvents	Concentration	Solution Preparation	Electrospinning Parameters	Post-Treatment
PET	TFA (HFIP):DCM = 1:1, 1:3, 1:4, 3:1, 4:1, 4.5:5.5, 3:7, 7:3	10–24 wt%	magnetic stirring at room temperature for 3 to 24 h	0.1–6.0 mL h^−1^, 13–24 kV, 4–25 cm, 0.2–0.8 mm needle	dried either in ambient air overnight/24 h or in a vacuum oven at ~50 °C for 2 h
PVC	DMF or THF:DMF = 1:1, 2:3, 4:1, 3:7, 3:2	4–15 wt%	magnetic stirring at room temperature or 40–100 °C for 2 to 24 h	0.2–3.0 mL h^−1^, 8.35–25 kV, 8–20 cm, 0.5–0.9 mm needle	dried either in ambient air 24 h or in a vacuum oven at 60 °C for 12 h
PS	DMF, DMF:chloroform = 3:1, DMF:acetone = 1:1	40 wt%	magnetic stirring at room temperature for 12 to 24 h	0.5–2.0 mL h^−1^, 12–22 kV, 10–20 cm, 0.5–0.8 mm	dried in ambient air or in a vacuum oven at 40–50 °C
PMMA	DMF or DCM:DMF = 4:1	7.5–21 wt%	magnetic stirring at room temperature overnight	0.5–4.0 mL h^−1^, 10–20 kV, 10–20 cm, 0.6–1.1 mm needle	dried in ambient air or under vacuum at 30–50 °C

Abbreviations: PET—polyethylene terephthalate; PVC—polyvinyl chloride; PS—polystyrene; PMMA—polymethyl methacrylate; TFA—trifluoroacetic acid; HFIP—hexafluoroisopropanol; DCM—dichloromethane; DMF—N,N-dimethylformamide; THF—tetrahydrofuran.

**Table 4:** Comparative assessment of waste-derived PET, PVC, PS, and PMMA for electrospun separator applications.

Criterion	PET	PVC	PS	PMMA
Electrospinnability	Well established; mature protocols (Table 2)	Viable after purification; partial-solubility constraint	Straightforward; highly soluble	Protocol established for commercial PMMA (Table 2); not yet tested on waste feedstock
Purification difficulty	Low; dissolves cleanly	High; centrifugation needed to remove CaCO_3_, plasticizers, stabilizers	Low; washed, dried, dissolved directly	Not established (no waste-derived study identified)
Solvent sustainability	Reliant on hazardous halogenated solvents (TFA, HFIP, DCM)	DMF/DMAc typical; no green alternative demonstrated in the studies reviewed here	Green alternative (d-limonene) already demonstrated	DMF-based (Table 2); no green alternative demonstrated; not yet tested on waste feedstock
Wettability	Poor pristine (WCA > 120°), improved below 40° via plasma/UV-ozone treatment	Poor pristine (WCA > 90°), improved via chlorine-substitution chemistry	Poor; demonstrated tunability has been directed toward greater water repellency, not less	No data
Thermal stability	No measurable shrinkage to ~150 °C, exceeding Celgard^®^ 2400	Not quantified against Table 1 shrinkage metric in waste-derived form	High intrinsic thermal stability (aromatic backbone); not benchmarked against Table 1	No data
Mechanical performance	Highly variable (1–62.5 MPa); often below the 70–140 MPa reported for Celgard^®^ 2400	Modulus comparable to Celgard (197 vs. 170 MPa); tensile strength far below (3.36 vs. 70–140 MPa)	Modulus brackets the Celgard range (114–1564 MPa)	No data

**Table 5:** Summary of Membrane Preparation and Electrochemical Testing.

Material	Preparation Method	Cell Configuration	Electrolyte	Characteristic Features	Electrochemical Performance	Refs
PET	Electrospinning	LFP||PET||Li	1 M LiPF_6_ in EC/DMC (1:1 vol.)	3D porous network structure with 330 nm diameter fibers; 40 mm thickness; TS: 12.0 MPa; strain-at-break: 42%; porosity: 89%; EU: 500%; IC: 2.27 × 10^−3^ S cm^−1^	The initial discharge capacity of 147.2 mAh g^−1^ with capacity retention of 139.9 mAh g^−1^ after 50 cycles	[30]
Recycled bottle-sourced PET/AlN	Electrospinning	NCM523||PET-AlN||Li	1 M LiPF_6_ in EC/EMC (1:1 vol.)	AlN nanoparticles encapsulated in PET micro-/nanoarchitecture fibres; porosity: 69.23%; electrolyte uptake (EU): 521.69%; WCA: 135°; ECA: ~0°	Discharge specific capacity of 238.2 mAh g^−1^ after 100 cycles at 0.5C; 179.2 mAh g^−1^ at 2C and 116.5 mAh g^−1^ at 4C	[33]
Al_2_O_3_/PVDF-HFP/PET	Coating and UV Curing	LFP|| Al_2_O_3_/PVDF-HFP/PET||Li	1 M LiPF_6_ in EC/DEC/DMC = 1:1:1, wt.	Thickness: 42 µm; porosity: 33.8%; average pore diameter: 0.6 µm; ECA 27.2°; Gurley: 486 s; EU: 69.1%; IC: 1.03 mS cm^−1^; TS: 15.5 MPa; strain-at-break: 12.7%; puncture strength: 155.3 g; shrinkage: none to 250 °C (2 h)	137.3 mAh g^−1^ (84.1% retention) after 300 cycles at 0.2C; 87.1 mAh g^−1^ (51.7%) at 5C rate.	[116]
PVDF-HFP/PET/PVDF-HFP	Electrospinning and solution casting	LFP||PVDF-HFP/PET/PVDF-HFP||Li	1 M LiPF_6_ in EC/DEC (1:1 vol.)	Sandwiched PET composite membrane with overall thickness of 100 μm; fiber diameter: 500–900 nm; EU: 282%, IC: 10^−3^ S cm^−1^, WCA: ~110°; ~8% shrinkage at 150 °C after 30 min; ECA: ~2.9°	132 ± 0.3 mAh g^−1^ at 10C at 55 °C; ~20% higher capacity retention after 200 cycles at 1C at 55 °C	[117]
PVA-Si/PET	Blade-coating	LFP||PET@PVA@SiO2||Li	1 M LiPF_6_ in EC/DEC/DMC (1:1:1 vol.)	Nonwoven PET fabric coated with a mixture of cross-linked PVA and silica nanoparticles (30 nm); thickness: 24 μm; EU: 306.6%; TS: ~25 MPa	The initial discharge capacity of 131 mAh g^−1^ with capacity retention rate of 99.1% after 100 cycles and 82.2% after 500 cycles at 1C (CE 98.1%); 101 mAh g^−1^ at 3C	[118]
PET GPEs (PEO-IL)	Solution casting and blade-coating	LFP||NW GPE||Li (full cell)	1 mM EMIM-TFSI	Macroporous network of interconnected fibres; thickness: 20 μm; TS: 53.2 MPa; IC: 4.08 × 10^−4^ S cm^−1^	A reversible capacity of 149.5 mAh g^−1^ after 70 cycles at 0.1C (~100% of its initial capacity) with CE of 96.7%	[119]
PET/PVDF	Electrospinning and hot-pressing	LMO||PET-PVDF||Li	1 M LiPF_6_ in EC/DEC/DMC (1:1:1 vol.)	Transverse and longitudinal TS: 13.70 and 34.85 MPa; porosity: 51.4%; EU: 124%; 1.1% shrinkage at 150 °C for 30 min; ionic conductivity (IC): 8.36 mS cm^−1^	The initial capacity of 112.55 mAh g^−1^ at 0.5C with capacity retention of 96.94% after 100 cycles; capacity retention of 87.4% at 4C rate	[120]
PET	Ion irradiation technology	LFP||ITE PET||Li	1 M LiPF_6_ in EC/DEC (1:1 vol.)	The uniformed vertical nanochannels with narrow channel size distribution (150–190 nm); Gurley value: 168 s 100 mL^−1^; WCA: 58.4°; electrolyte contact angle (ECA): 16.5°; TS: 67.6 MPa; shrinkage less than 3% at 180 °C	Discharge specific capacity of 110.8 mAh g^−1^ at 1C; capacity retention ratio of 80.23% after 300 cycles at 1C	[121]
PVDF/PET	Thermally Induced Phase Separation	LFP||SSCSs||Li	1 M LiPF_6_ in EC/DEC/DMC (1:1:1 vol.)	The sandwich-structured composite separator with an anisotropic pore structure; ECA: 16.7°; EU: 259.7 ± 3.2%; TS: 10.0 ± 0.6 MPa; strain-at-break: 40%; IC: 0.98 ± 0.09 mS cm^−1^ at 25 °C and 0.22 ± 0.03 mS cm^−1^ at 200 °C for 0.5 h	The initial discharge capacity of 150.0 mAh g^−1^ with capacity retention of 95.3% after 100 cycles	[81]
PET/PVDF	Electrospinning and blade-casting	NMC532||nPPV-SPE||Li ASSLMBs (full cell)	nPPV-SPE	Bilayer nonwoven PET microfiber/PVDF nanofiber composite; TS: 15.8 MPa; strain-at-break: 47.7%	The initial discharge capacity of ~155 mAh g^−1^ with capacity retention of ~85% after 60 cycles	[80]
PVDF–PVC	Electrospinning	LFP||PVDF–PVC PEs||Li	1 M LiClO_4_ in PC/EC (1:1 vol.)	PVDF–PVC (8:2, *w*/*w*) polymer electrolytes; fiber diameter: 624 nm; porosity: 87%; EU: 290.5%; IC: 2.25 × 10^−3^ S cm^−1^; Xc: 30.0%; thickness: ~0.1 mm	The discharge capacity of 130.8 mAh g^−1^ after 50 cycles with 90.1% capacity retention	[60]
CA/PS	Solution-based blow spinning	ACF-PEDOT ||CA/PS||ACF-PEDOT (Supercapacitor)	Simulated sweat solution	Cellulose acetate and polystyrene based composite separator with dense zone and dispersed fibers zone; pore size: 15 μm; porosity: 59%; EU: 251%; thickness: 1.2 mm	Specific capacitances: 33 Fg^−1^ at 5 mVs^−1^; washing stability up to 30 cycles and capacitance retention of 95% up to 1000 cycles	[54]
PMMA gel polymer	In situ crosslinking polymerization	NMC111||PMMA GPE||graphite	PMMA GPE containing 0.9 M LiTFSI and 0.1 M LiBOB solution in EC/PC (7:3 vol.)	Crosslinked PMMA gel electrolyte; IC: 3.9 mS cm^−1^ at 20 °C; stress and shear moduli: 66.7 Pa and 2.66 MPa	Graphite/NMC cells with 2.1 mA h cm^−2^ capacity for hundreds of cycles; 80% capacity loss occurs only after 390 cycles	[64]
PP/PMMA	Suspension polymerization	S||PP/PMMA||Li	1 M LiTFSI in DME/DOL (1:1 vol.)	Double-layered separator composed of PMMA microspheres coated on the surface of the macroporous PP matrix; thickness: ~1.5 μm	The initial discharge capacity of 1100 mAh g^−1^ at 0.1 mA cm^−2^ with capacity retention of 613.40 mAh g^−1^ after 100 cycles (CE 98–100%); 387 mAh g^−1^ at 5C	[61]
PVDF/PMMA	Non-solvent-induced phase separation	LiCoO_2_||PVDF/PMMA||Li	1 M LiPF_6_ in EC/EMC/DMC (1:1:1 vol.)	A gel polymer electrolyte based on the blending membranes; IC: 2.18 mS cm^−1^, ECA: 14.53°; 37.8% shrinkage rates at 160 °C for 30 min	130.7 mAh g^−1^ after 200 cycles at 1C (CE ~100%); 133.3 mAh g^−1^ at 4C	[62]

Abbreviations: ITE—ion irradiation technology; LFP—lithium iron phosphate; NCM523—LiNi_0.5_Co_0.2_Mn_0.3_O_2_; LMO—lithium manganese oxide; GPE—gel polymer electrolyte; SPE—solid polymer electrolyte; NW—nonwoven; ASSLMB—all-solid-state lithium metal battery; EU—electrolyte uptake; IC—ionic conductivity; ECA—electrolyte contact angle; CE—Coulombic efficiency; PP—polypropylene; PVDF—poly(vinylidene fluoride); PVDF-HFP—poly(vinylidene fluoride-hexafluoropropylene); PVA—poly(vinyl alcohol); PEO—poly(ethylene oxide); IL—ionic liquid; CA—cellulose acetate; ACF—activated carbon fiber; PEDOT—poly(3,4-ethylenedioxythiophene); EC—ethylene carbonate; DEC—diethyl carbonate; DMC—dimethyl carbonate; EMC—ethyl methyl carbonate; PC—propylene carbonate; DME—dimethoxyethane; DOL—1,3-dioxolane; LiPF_6_lithium hexafluorophosphate; LiClO_4_—lithium perchlorate; LiTFSI—lithium bis(trifluoromethanesulfonyl)imide; LiBOB—lithium bis(oxalato)borate; EMIM-TFSI—1-ethyl-3-methylimidazolium bis(trifluoromethanesulfonyl)imide.

## Data Availability

No new data were created or analyzed in this study. Data sharing is not applicable to this article.
